# Nystagmus with Report of Some Rare Cases

**Published:** 1912-12

**Authors:** A. W. Stirling

**Affiliations:** Atlanta, Ga.; Ophthalmic Surgeon to Grady Hospital, Etc.


### Journal-Record of Medicine

Successor to Atlanta Medical and Surgical Journal, Established 1855
and Southern Medical Record, Established 1870.

**OWNED BY THE ATLANTA MEDICAL JOURNAL CO.**

**Published Monthly**

*Official Organ Fulton County Medical Society, State Examining Board, Presbyterian Hospital, Atlanta, Birmingham and Atlantic Railroad Surgeons' Association, Chattahoochee Valley Medical and Surgical Association, Etc.*

EDGAR G. BALLENGER, M. D., Editor.
BERNARD WOLFF, M. D., Supervising Editor.
A. W. STIRLING, M. D., C. M., D. P. H., J. S. HURT, B. Ph., M. D.
GEO. M. NILES, M. D., W. J. LOVE, M. D., (Ala.); Associate Editors.
E. W. ALLEN, Business Manager.

COLLABORATORS

Dr. W. F. WESTMORLAND, General Surgery.
F. W. McRAE, M. D., Abdominal Surgery.
H. F. HARRIS, M. D., Pathology and Bacteriology.
E. B. BLOCK, M. D., Diseases of the Nervous System.
MICHAEL HOKE, M. D., Orthopedic Surgery.
CYRUS W. STRICKLER, M. D., Legal Medicine and Medical Legislation.
E. C. DAVIS, A. B., M. D., Obstetrics.
E. G. JONES, A. B., M. D., Gynecology.
R. T. DORSEY, Jr., B. S. M. D., Medicine.
L. M. GAINES, A. B., M. D., Internal Medicine.
GEO. C. MIZELL, M. D., Diseases of the Stomach and Intestines.
L. B. CLARKE, M. D., Pediatrics.
EDGAR PAULIN, M. D., Opsonic Medicine.
THEODORE TOEPEL, M. D., Mechano Therapy.
R. R. DALY, M. D., Medical Society.
A. W. STIRLING, M. D., etc., Diseases of the Eye, Ear, Nose and Throat.
BERNARD WOLFF, M. D., Diseases of the Skin.
E. G. BALLENGER, M. D., Diseases of the Genito-Urinary Organs.

VOL. LIX. DECEMBER 1912 No. 9

### NYSTAGMUS WITH REPORT OF SOME RARE CASES.

BY A. W. STIRLING, M. D., M. B., C. M., (EDIN.) D. P. H. (ENG.), ATLANTA, GA.

*Ophthalmic Surgeon to Grady Hospital, Etc.*

Before describing a few somewhat peculiar cases nystagmus which have recently come under my notice, perhaps it would be well to say a word concerning the general aspects of the disease itself. It is hardly necessary to remind you that the ocular normal movements are unaffected, that the oscillations, which may vary from a very few to 350 or more a minute, may be horizontal, vertical, rotary or a mixture of these; and that they are usually binocular, only some 60 cases having been recorded as affecting one eye alone; that they cease during sleep and that cases are either congenital (or at least early infantile) or acquired. Associated with those which appear in early infancy is usually to be found some lesion of the media, or fundus, such as malformations, absence of pigment, opacities of cornea orlens. In a few of these cases no lesion can be discovered, though in some such one or more near relatives have been albinotic. When the eyes are actually blind there is no nystagmus.

A special and peculiar form is that which is acquired in infancy, after the power of fixation has been developed and is associated with movements of the head—spasmus nutans—and which may disappear with time and sometimes in one eye before the other. In the congenital or early infantile cases it is evident that the principal cause is inability to fix with the normal power of the fovea. Central macular fixation is not normally acquired till some weeks after birth, and in disease the macula either may have been destroyed or may have no opportunity, on account of medial opacities, to bring its peculiar potentiality of acute vision into play. The eye therefore continues to use any part of the retina indifferently, though it would appear as if it were dissatisfied and continually were in search by its movements of a clearer and more normal vision. It has, however, also been suggested that the cause of the intermittent motion may be analogous to the interrupted current of an electric battery.

*Acquired Nystagmus*, besides its interest as a pathological study, is of considerable practical importance, because, as Snell has shown, a nystagmic coal miner cannot be relied upon to see the so-called "cap" upon the miner's lamp which is the trusted warning of the absence of fire-damp. For while congenital nystagmus does not give an appearance of motion to objects looked at, the acquired form does. It is therefore of more than passing interest in coal mining districts. Miners' nystagmus may be horizontal and vertical but there is always a rotary motion in its make-up.

As to the etiology: It used to be considered as settled that miners' nystagmus is one of the "occupation neuroses" and due to the cramped position and the upward gaze of the miner. But if that were so, why is it that other miners besides those in the coal fields are not affected? A number of theories have been advanced in more recent times, e. g. (2) (Edridge-Green) it may be caused by the necessity for movement of the eye in order that the fovea, or central sensitive spot, may see,, because the fovea is blind when there is no visual purple in it, and the diffusion of the visual purple into the fovea is carried either by light falling on the adjacent portion of the retina (which does not happen in a dark mine) or by ocular movements, (3) (Llewellyn) or; it may be due to a condition of imperfect centripetal impulses (imperfect fixation, disturbance of equilibrium, etc.) the intimate connection between the centers governing the associated movements of the eyes being lost and inco-ordinate movements ensuing." (4) Another idea (Rutten) is that nystagmus is a reflex act compensatory to the general unnatural position of the body. (5) Yet another writer (Weekers) ,while believeing that no satisfactory explanation of miner's nystagmus has yet been supplied, draws attention to the frequency with which night blindness is associated with it.

Besides miners, others may suffer from the acquired form of these symptoms, such as patients with disseminated scelrosis cerebellar disease, and affections of the semi-circular canals.

It may also be as well to remember the necessity of distinguishing true nystagmus from that comparatively slow and not so purely rhythmic nystagmoid jerking which may occasionally be elicited in the normal eyes of fatigued persons when they look far to one side.

The following cases may be of a certain amount of general interest. The first is one of voluntary nystagmus. I published it in the Ophthalmoscope of London, in February of this year, and the strange coincidence happened that in the same issue appeared another case by Major Elliott of the Indian Medical Service. These two cases evidently incited Dr. Waddy of Oxford to collect all the published cases of voluntary nystagmus which he coud discover, and these appeared in the same journal last June to the number of 17. The most curious of these, one of what he calls the freak type, because the patient made a living in Germany by exhibiting his peculiarities, which Dr. Waddy had himself witnessed, consisted in the voluntary production of a one-eyed horizontal nystagmus, a voluntary dilatation of the pupils unassociated with convergence or divergence of the globe, and the inhibition of the right external rectus whereby he could prevent the right eye from crossing beyond the right vertical plane, while the left cornea crossed to the caruncle.

All the cases were horizontal except one.

The following is my case of voluntary nystagmus:

Mrs. J. D. L.—Age 24. Came complaining of pain in and over the eyes. The refraction was as follows: R V—6-12 HM2D 6-9 no cyl. L V 6-60 H M—4:5 D=6-18 no cyl. The fundus was normal. There never had been strabismus. There were $5^{\circ}$ of latent convergence, and the right eye looked down $4^{\circ}$. But the interesting point in the case lay in the fact that ever since childhood she had been able at will to produce a voluntary nystagmus. The ocular excursions were too rapid to count, almost as rapid as one can shake one's hand from side to side, horizontal, and perhaps about one millimeter in length.

I might also cite the case of Voluntary Nystagmus by R. H. Elliott, M. D., Lond., F. R. C. S., Eng.; Major I. M. S.; Superintendent government Ophthalmic Hospital, Madras, India.

'In the leisure of a board-ship passage out to India recently, a fellow passenger showed me a curious eye-trick, of which he seemed not a little proud. He is a chemical expert, with the degree of Ph. D. of Heidelberg, and it naturally follows that he is very highly educated, and a good observer; he is an active man of good physique and of exceptional powers of endurance; his age is 30; he has, according to his own statement, exceptionally keen distant and reading sight. I had no means of testing this by types, but he could pick up objects on the horizon quickly and well. He has never had headaches or any other signs of eye-strain; he has been able to produce nystagmus voluntarily as long as he can remember. To do so he stares straight in front of him, and within a second or two the phenomenon begins. It is a binocular, horizontal, oscillatory movement; the amplitude is large and the movements are too rapid to count. There has never been a movement in any direction other than the horizontal; the oscillations ceases as soon as, or very soon after the voluntary effort is relaxed. When he wishes to produce the nystagmus, he is conscious of a sense of effort which he refers to the situation of the external recti-muscles of both eyes. During the nystagmus, objects appear to be blurred in the sense that they are drawn out sideways; a feeling of tiredness, which is becoming more marked now, as he grows older, attends the production of the phenomenon. On several occasions of recent years he has noticed that the nystagmus has commenced spontaneously; this has occurred when he has been overtired; he has been able to control it, almost at once, on such occasions. On the other hand, it is easier for him to produce the nystagmus when he is fresh than when he is tired.

I can only find the scantiest references in some of the larger text-books to the existence of the phenomenon of voluntary nystagmus, and I cannot remember to have met with a similar case previously. I therefore publish these notes although they are necssarily scanty and somewhat unsatisfactory."

The following case I reported at the Southern Medical Association in 1909:

For the last two years, only on *closing the lids,* the eyes of a lady, aged 20 years, have made lateral nystagmic movements, 85 to 100 double motions a minute. This stops during sleep; and also for about one minute she can hold the closed eyes still by an effort, during which, however, the upper lids constantly quiver. On looking up, with the eyes open, there is some tendency to a similar movement, but it is not constant. Refraction is about 1 D cyl.; axis oblique=6-5. There is a latent divergence of 3°. The patient has always been rather nervous and "jumpy," has floating kidney, and suffers during menstruation and from rheumatic joints.

### Nystagmus With Shaking Head

Mr. V. T. B.—Age 76. Wants glasses. His vision has always been poor. Under homatopine and cocaine: R V 6-60 with 1 D Dsp=6-60; L V= with 75 Dsp.—6-24 Right pupil does not act to light but does to accom. and consensually. Left pupil is normal. Bothh discs are rather pale and waxy. No retinitis pigmentosa of the usual order; some mottling round the discs. On making patient look to one side and up a lateral nystagmus is developed. On inquiry it then appears that out of elevn children in the family one brother and one sister had nystagmus. Their sight was "good" and they were not albinos. These two also developed a constant, quick jerking to and fro of the head "spasmus-nutens" which they could stop at will. A daughter of the afflicted brother (she is now 68) also has nystagmus and a similar shaking of the head.

### Almost Albinos, Without Nystagmus, But With Poor .. Vision

In the following two cases are associated a state verging upon albinism without nystagmus, but with a low acuity of vision:

Miss L. G.—Age 29. Is nearly an albino, but her eyes are distinctly blue. Her brother is an albino with nystagmus. Her vision is as follows under homatopine and cocaine: R V—6 36 with 25 Dsp. + 3 D cyl. ax. 50 6 18 partly L V=6 60 with +5 Dsp.=3 D cyl. ax. 105°=6 12 party. Two days later ordered cylinders partly 6 12. The fundi were very pale.

Miss M. B.—Age 7. Has always had poor sight. Her fundi contain no pigment. Had very pale hair as a baby, but her eyes were always blue. Under a mydriatic: R V=6-60 with 7 Dsp. 6-60. L V=6-60 9 Dsp. 6-60.

### Albinsm With Permanent Foramen Ovale and Nystagmus

The appearance of the following patient is remarkable, seeing that in her skin and mucous membranes are combined the coloring of an *albino* and that of the blood in one in whom the *foetal opening between the two sides of the heart has never closed.* Slight nystagmus is also present, but is now much less marked than formerly. There is a little blue color in the irides.

R V=6-60 with +6 O Dsp. 6-60. L V=6-60 with +5-5-5 D 6-60. There is convergent concomitant strabismus.

The cases here recorded belong, as you see, to different categories. The rapidity of movement in voluntary nystagmus might suggest that there is a natural tendency to nystagmus in a developed seeing eye, which is normally obliterated by the fact of central vision, and that this inhibition, can be performed voluntarily by a few isolated persons. The association of nystagmus with shaking head, which is a better known phenomenon, may perhaps somewhat resemble other associated movements in which the eye has a part, as in winking along with certain motions of the mouth. The rhythmic activity of nerve centers is the so-called explanation of the phenomenon.

The constant association of nystagmus with albinism is interesting, though more simple, because the lack of ocular pigment plainly interferes with any well defined central vision. The borderland cases, which I quote, are, I think, instructive. In these the patients are sufficiently nearly albinotic to have very defective vision, but not so defective in pigment as to be nystagmic.

### Discussion by A. W. S.

I was admitted in Dr. Nelson's cases, especially in the result of the decompression operation as I presume the operation virtually was. The theory he mentions as explanatory of nystagmus appears to me hardly to hold water, because the imptus sent from each side of the brain goes to both eyes, and therefore could hardly produce the intermittent action of which he spoke. Besides, is there any ground for such a theory in the facts of congenital nystagmus?

Dr. Lokey's suggestion that the state of the inner ear might account for miner's nystagmus is ingenious, but at least in my experience aural nystagmus is usually associated with dizziness, and so far as I am aware this symptom is not one complained of by persons suffering from miners' nystagmus.

### DISCUSSION BY DR. R. M. NELSON

Among the causes of nystagmus, Dr. Stirling failed to mention cerebellar disease. Many now consider nystagmus an important diagnostic sign in disease of the cerebellum. Dr. W. L. Ballenger in the latest edition of his text-book "Diseases of the Nose, Throat and Ear," enters several paragraphs to its discussion in this connection.

I have seen two patients with clinical diagnosis of cerebellar involvement, one of whom had typical laternal nystagmus, the other showing no nystagmus of any kind. Both cases were seen by me during my service of six (6) years (1906-1912) as Chief of Eye, and Ear Clinic, Colon Hospital, Cristbal, Canal Zone, Panama. As these cases appear of interest in this discusson I wlll give some details of both. I quote largely from hospital Panama. As these cases appear of interest in this discussion, I will give some details of both.

Case No. 1, May 3rd, 1907, Manuel M., Spanish-American, referred by Dr. James of medical staff for opthlmoscopic examination. No detailed history obtainable, as patient almost comatose and only statement secured from him was that he had had convulsions. The chart showed a subnormal temperature 97° F. and lower, with a pulse rate as low as 30 to 40 a minute. Examination of the eyes under atropine showed the outlines of the optic nerve (in each eye) indefinite and appearing as though the nerve generally merged into the surrounding retna; veins congested, swollen, tortuaus, arteries small, apparently contracted or lessened in colibr. The optic nerve disk was elevated above the surrounding retina with perceptible dipping outward and backward of the vessels. In other words it showed the typical appearance of bilateral papillitis, or "choked disc." There was present convergent strabismus, and rather typical lateral nystagmus. The advisability of trephining the skull and exploring for brain abscess or tumor was considered. On May 4th it was observed that the patient frequently placed his hand to his right ear and mastoid region. Examination of ears showed as follows: Right ear drum whitish, dull, ear drum bulging. Incision followed by evacuation thick, yellowish pus, with foul odor. The patient was in such a condition of stupor as to be practically insensible to pain. So no tenderness to be made out over mastoid. No odematous swelling soft tissues mastoid, though in fact such soft tissue ocedemae not often found in adults with mastoiditis. Left ear showed evidence of moderate catarhal otitis media acute. Incision left ear drum not indicated. May 6th mastoid operation performed prior to trephining over cerebellar region. The mastoid cells were found full of pus, and some necrosed bone was removed. Trephining over cerebellum revealed cerebellum abscess. At least one ounce (℥I) of thick, yellowish pus was evacuated. After the operation the patient never roused from the condition of stupor or coma. The temperature rose gradually until it had reached over 106° F., when death occurred within ten hours after operation. Recovery after cerebellar abscess is very rare. An autopsy was held by Dr. Norman Williamson, Pathologist Colon Hospital, and it was shown very clearly how the infection had traveled from the middle ear through the internal ear (labyrinth, etc.) and internal auditory meatus and to the posterior fossa of the skull to the cerebellum. Pus was found in the body of the sphenoid bone. Some catarrhal inflammation was present in the left middle ear, and also in both maxillary and frontal sinuses. Petechial hemorrhages were present in the intestines and spleen, and there was fatty degeneration of the liver, all indicative. Dr. Williamson said, of death being due to general sepsis. This case is described at length as an example of how a neglected infection of the middle ear may find its way ultimately to the brain itself. And also because nystagmus was so well marked in this patient. McKernon of New York, states that not less than 30 per cent. of brain abscesses are caused by a suppurative otitis media.

Case No. 2. A white American was referred to me for ophthalmicroscopic examination for "checked disk." He had been having severe occipital pain, dizziness, vomiting, tinnitus aurium, vision slightly less acute than usual. Bilateral elevation of nerve heads of exactly three diopters was found and other appearances of "choked disc." Similar exploration of cerebella region to that described under Case 1, was done but thorough search failed to find any cerebellar or other brain tumor. Dr.

Harvey Cushing has stated that not more than 40 or 50 per cent of cerebellar tumors can be found at operation when actually present. Hence not finding is not definite negative proof.

After operation this patient showed marked and rapid improvement. All the symptoms including the "choked disk" and dimness of vision rapidly disappeared. Within four weeks was discharged from hospital. I met him at intervals for several months and he was always enthusiastic in describing his good health and freedom from symptoms. However, after about five or six months he re-entered hospital, with return of his old symptoms. Was referred to me for ophthalmicroscopic examination, "choked disk" had re-appeared, and rapidly assumed same degree elevation. Patient's condition grew steadily worse and finally death took place. At no time while under our observation did this patient show nystagmus. Though examinations of ears showed them to be normal. I may add that Barany's method of testing labyrithive reactions with hot and cold water irrigation of ear was not used. If used, useful information might possibly have been obtained. In this case I can give no findings, as I know of no post mortem being performed.

Professor Fuchs (Fuchs), Professor Ophthalmatogy University of Vinenna, mentions an interesting theory as to cause of nystagmus, which he states will explain even the cases of voluntary, disassociated and other rare forms of nystagmus. According to this theory in normal fixation motor impulses from the two sides of the brain are simutaneous and hence act as check to each other and the eyes are still. Whereas in certain conditions the motor impulses from two sides of brain leave alternately instead of simultaneously—and the oscillatory movements of eyelids result.

In occupational, optical, and aural forms of nystagmus no lesions of brain found. In nystagmus in cases of disease of nervous system lesions have been found after death in corpora quadrigemina corpus storiatum restiform bodies, cerebellum and medulla. Nystagmus is present in about 25 per cent. of cases of multiple sclerosis. It is found in syringo myetia though not always.

